# *In Vitro* Activity of Cefiderocol against Extensively Drug-Resistant Pseudomonas aeruginosa: CANWARD, 2007 to 2019

**DOI:** 10.1128/spectrum.01724-22

**Published:** 2022-06-27

**Authors:** James A. Karlowsky, Andrew J. Walkty, Melanie R. Baxter, Heather J. Adam, Philippe R. S. Lagacé-Wiens, Frank Schweizer, Denice Bay, Joseph P. Lynch, Michael R. Mulvey, George G. Zhanel

**Affiliations:** a Department of Medical Microbiology and Infectious Diseases, Max Rady College of Medicine, University of Manitoba, Winnipeg, Manitoba, Canada; b Clinical Microbiology, Diagnostic Services, Shared Health, Winnipeg, Manitoba, Canada; c Department of Chemistry, Faculty of Science, University of Manitoba, Winnipeg, Manitoba, Canada; d Division of Pulmonary and Critical Care Medicine, Clinical Immunology, and Allergy, David Geffen School of Medicine at UCLA, Los Angeles, California, USA; e Public Health Agency of Canada—National Microbiology Laboratory (PHAC-NML), Winnipeg, Manitoba, Canada; University of Texas MD Anderson Cancer Center

**Keywords:** antibacterial, bacterial, cephalosporin, susceptibility

## Abstract

Cefiderocol was evaluated by broth microdilution versus 1,050 highly antimicrobial-resistant Pseudomonas aeruginosa clinical isolates from the CANWARD study (2007 to 2019). Overall, 98.3% of isolates remained cefiderocol susceptible (MIC, ≤4 μg/mL), including 97.4% of extensively drug-resistant (XDR) (*n *= 235) and 97.9% of multidrug-resistant (MDR) (*n *= 771) isolates. Most isolates testing not susceptible to ceftolozane-tazobactam, ceftazidime-avibactam, and imipenem-relebactam remained susceptible to cefiderocol. *In vitro* data suggest that cefiderocol may be a treatment option for infections caused by MDR and XDR P. aeruginosa.

**IMPORTANCE** After testing cefiderocol against a large collection of clinical isolates of highly antimicrobial-resistant Pseudomonas aeruginosa, we report that cefiderocol is active versus 97.4% of extensively drug-resistant (XDR) and 97.9% of multidrug-resistant (MDR) (*n* = 771) isolates. These data show that cefiderocol may be a treatment option for infections caused by MDR and XDR P. aeruginosa.

## OBSERVATION

Pseudomonas aeruginosa is an important nosocomial pathogen (1). It is frequently implicated as a cause of hospital-acquired urinary tract infections, pneumonia, wound/surgical site infections, and bacteremia, as well as infections among immunocompromised patients and those with burns (1). Treatment of infections caused by P. aeruginosa can be problematic, as this pathogen demonstrates intrinsic resistance to many different antimicrobials (2, 3). Additionally, P. aeruginosa clinical isolates can acquire resistance to the limited number of antimicrobials that do possess antipseudomonal activity, leaving clinicians with few therapeutic options (1, 3). Acquired beta-lactam resistance among P. aeruginosa may be mediated by a variety of mechanisms, including derepression of AmpC, acquisition of metallo-beta-lactamases, reduced antimicrobial permeability, and overexpression of efflux pumps (1, 4).

Cefiderocol is a novel parenteral siderophore cephalosporin that utilizes the bacterial iron uptake system for entry into cells (5). It demonstrates *in vitro* activity against a wide range of Gram-negative bacteria, including P. aeruginosa (5). Cefiderocol is resistant to hydrolysis by the chromosomal AmpC found in P. aeruginosa, and it has a low propensity for induction of this enzyme (6). It also demonstrates stability versus clinically relevant carbapenemase enzymes, including many metallo-beta-lactamases (7). Additionally, overproduction of the MexAB-OprM efflux pump and loss of OprD in P. aeruginosa do not appear to adversely affect the *in vitro* activity of this antimicrobial (8). These properties make cefiderocol an appealing option for the treatment of infections caused by multidrug-resistant (MDR) and extensively drug-resistant (XDR) P. aeruginosa isolates.

Cefiderocol has demonstrated comparable efficacy to carbapenems in the treatment of complicated urinary tract infections (APEKS-cUTI) and nosocomial pneumonia (APEKS-NP) (9, 10). However, in clinical practice, this antimicrobial may be reserved for patients with infections caused by difficult-to-treat pathogens, including P. aeruginosa. Several case reports and one case series have been published describing the successful use of cefiderocol for the treatment of infections caused by MDR or XDR P. aeruginosa (11–13). The purpose of this study was to evaluate the *in vitro* activity of cefiderocol versus a collection of highly antimicrobial-resistant clinical isolates of P. aeruginosa obtained from patients admitted to or evaluated at hospitals in Canada between 2007 and 2019.

The P. aeruginosa clinical isolates included here were collected as part of the CANWARD study (January 2007 to December 2019) (14). CANWARD is an ongoing national Public Health Agency of Canada (PHAC)/Canadian Antimicrobial Resistance Alliance (CARA) partnered surveillance study designed to assess antimicrobial resistance among bacterial pathogens recovered from patients receiving care at hospitals in major population centers across Canada (http://www.can-r.ca/). On an annual basis, each participating center was asked to submit clinical isolates (consecutive, one per patient per infection site) from blood, respiratory, urine, and wound infections. The study isolates were shipped to the coordinating laboratory (Health Sciences Centre, Winnipeg, Canada), where their identities were confirmed by colonial appearance, spot testing (15), and/or matrix-assisted laser desorption ionization–time of flight (MALDI-TOF) mass spectrometry (Bruker Daltonics, Billerica, MA, USA). The isolates evaluated in this study were a subset of all P. aeruginosa isolates recovered in CANWARD and were selected for this study if they were XDR, MDR, or not susceptible to any one of the antipseudomonal agents tested by CANWARD. MDR and XDR isolates were defined as those testing not susceptible to ≥3 (MDR) or ≥5 (XDR) of the following: antipseudomonal cephalosporins (ceftazidime or cefepime), antipseudomonal carbapenems (meropenem or imipenem), antipseudomonal penicillins (piperacillin-tazobactam), fluoroquinolones (ciprofloxacin), aminoglycosides (gentamicin or tobramycin), and colistin (only resistant isolates were included in the MDR and XDR definition) (16).

Following two subcultures from frozen stock, the *in vitro* activity of cefiderocol and relevant comparators was determined by broth microdilution following the Clinical and Laboratory Standards Institute (CLSI) reference method (17). In-house-prepared 96-well broth microdilution panels with cation-adjusted Mueller-Hinton II broth (BD BBL; Becton, Dickinson and Company, Sparks, MD) were used for antimicrobial susceptibility testing. Cefiderocol was tested in chelating resin-treated iron-depleted cation-adjusted Mueller-Hinton II broth (2). All antimicrobial agents were acquired as laboratory-grade powders from their respective manufacturers or from a commercial source. MICs were interpreted using 2022 CLSI breakpoints (2). For cefiderocol, the CLSI interpretive criteria for P. aeruginosa are as follows: susceptible, ≤4 μg/mL; intermediate, 8 μg/mL; and resistant, ≥16 μg/mL (2).

In total, 1,050 P. aeruginosa isolates were evaluated in the current study. Specimen sources of the isolates were 74% respiratory, 12% wounds, 10% blood, and 4% urine. The *in vitro* activity of 13 antimicrobial agents tested against the isolates is provided in Table 1. Overall, 98.3% of all isolates were susceptible to cefiderocol, with 50% of isolates (MIC_50_) inhibited at a cefiderocol concentration of 0.5 μg/mL and 90% of isolates (MIC_90_) inhibited at a cefiderocol concentration of 2 μg/mL. The *in vitro* activity of cefiderocol and key comparators versus P. aeruginosa isolates not susceptible to common antipseudomonal antimicrobials is presented in Table 2. Cefiderocol MIC distributions for P. aeruginosa isolates, stratified by resistance phenotype, are provided in Table 3. The MIC_90_ of cefiderocol was 2 μg/mL or 4 μg/mL for all subsets not susceptible to other antipseudomonal antimicrobials; 97.4% of XDR (*n *= 235; MIC_50_, 0.5 μg/mL; MIC_90_, 4 μg/mL) and 97.9% of MDR (*n *= 771; MIC_50_, 0.5 μg/mL; MIC_90_, 2 μg/mL) P. aeruginosa isolates were cefiderocol susceptible. The majority of isolates testing not susceptible to ceftolozane-tazobactam, ceftazidime-avibactam, and imipenem-relebactam remained susceptible to cefiderocol (95.7%, 96.5%, and 98.7%, respectively).

**TABLE 1:** *In vitro* activity of cefiderocol and comparator antimicrobial agents against selected antimicrobial-resistant P. aeruginosa isolates cultured from specimens of Canadian patients from 2007 to 2019 (CANWARD surveillance study)

P. aeruginosa antimicrobial agent	MIC (μg/mL)				MIC interpretation (%)		
	MIC_50_	MIC_90_	MIC range		Susceptible	Intermediate	Resistant
Cefiderocol	0.5	2	≤0.06 to 32		98.3	1.1	0.6
Ceftolozane-tazobactam	2	8	0.25 to >64		86.9	6.1	7.0
Ceftazidime-avibactam	8	16	0.5 to >16		73.0	NA^*a*^	27.0
Imipenem-relebactam	1	4	≤0.03 to >32		77.7	12.8	9.5
Piperacillin-tazobactam	32	256	≤1 to >512		29.6	36.5	33.9
Meropenem	8	32	0.25 to >32		29.0	15.8	55.2
Imipenem	8	32	0.12 to >32		30.9	11.1	58.0
Cefepime	16	32	1 to >64		35.9	39.0	25.1
Ceftazidime	16	>32	2 to >32		36.6	19.7	43.7
Ciprofloxacin	2	16	≤0.06 to >16		28.6	18.3	53.1
Gentamicin	4	>32	≤0.5 to >32		65.0	13.9	21.1
Tobramycin	1	64	≤0.5 to >64		81.2	2.3	16.5
Colistin	1	2	0.12 to >16		NA^*b*^	95.9	4.1

a NA, not applicable; an MIC intermediate breakpoint is not defined for ceftazidime-avibactam.

b NA, not applicable; an MIC susceptible breakpoint is not defined for colistin.

**TABLE 2:** *In vitro* activity of cefiderocol and selected comparators against P. aeruginosa isolates with antimicrobial-resistant phenotypes

P. aeruginosa phenotype (no. of isolates)	Cefiderocol			Ceftolozane-tazobactam			Ceftazidime-avibactam^*a*^			Imipenem-relebactam	
	MIC_50_/MIC_90_ (μg/mL)	S/I/R^*b*^ (%)		MIC_50_/MIC_90_ (μg/mL)	S/I/R (%)		MIC_50_/MIC_90_ (μg/mL)	S/I/R (%)		MIC_50_/MIC_90_ (μg/mL)	S/I/R (%)
All isolates (1,050)	0.5/2	98.3/1.1/0.6		2/8	86.9/6.1/7.0		8/16	73.0/NA/27.0		1/4	77.7/12.8/9.5
XDR^*c*^ (235)	0.5/4	97.4/2.6/0		4/32	66.8/11.9/21.3		8/>16	57.0/NA/43.0		4/16	49.8/24.7/25.5
MDR^*d*^ (771)	0.5/2	97.9/1.4/0.6		2/8	82.5/8.3/9.2		8/16	64.6/NA/35.4		2/8	71.2/15.8/13.0
Ceftolozane-tazobactam-NS^*e*^ (138)	1/4	95.7/3.6/0.7		16/>64	0/47.1/52.9		16/>16	34.8/NA/65.2		2/32	53.6/14.5/31.9
Ceftazidime-avibactam-R (283)	1/4	96.5/2.1/1.4		2/64	68.2/12.7/19.1		16/>16	0/NA/100		2/8	61.5/20.1/18.4
Imipenem-relebactam-NS (234)	0.5/4	98.7/1.3/0		2/16	72.6/10.7/16.7		8/>16	53.4/NA/46.6		4/16	0/57.3/42.7
Piperacillin-tazobactam-NS (739)	0.5/2	97.8/1.5/0.7		2/8	82.1/8.7/9.2		8/16	62.4/NA/37.6		2/8	72.0/15.6/12.4
Meropenem-NS (745)	0.5/2	97.7/1.6/0.7		2/8	83.8/7.3/8.9		8/16	67.5/NA/32.5		2/8	68.6/18/13.1
Imipenem-NS (726)	0.5/2	98.1/1.4/0.6		2/8	84.2/7.1/8.7		8/16	70.8/NA/29.2		2/8	67.8/18.4/13.8
Cefepime-NS (673)	0.5/2	97.6/1.5/0.9		2/16	80.4/9.3/10.3		8/16	60.0/NA/40.0		2/8	69.5/16.7/13.8
Ceftazidime-NS (666)	0.5/4	97.6/1.5/0.9		2/16	79.4/9.8/10.8		8/16	58.3/NA/41.7		2/8	70.6/16.3/13.1
Ciprofloxacin-NS (750)	0.5/2	98.1/1.1/0.8		2/8	83.6/7.5/8.9		8/16	69.3/NA/30.7		2/8	72.7/15.0/12.3
Gentamicin-NS (368)	0.5/2	98.4/1.6/0		2/16	76.1/9.5/14.4		8/16	70.4/NA/29.6		2/8	61.4/19.9/18.7
Tobramycin-NS (197)	0.5/2	97.5/2.5/0		2/64	66.5/12.2/21.3		8/16	67.0/NA/33.0		2/16	52.8/23.3/23.9
Colistin-NS (43)	1/4	97.7/2.3/0		2/64	69.8/6.9/23.3		8/>16	58.1/NA/41.9		2/8	72.1/13.9/14.0

a NA, not applicable; an MIC intermediate breakpoint is not defined for ceftazidime-avibactam.

b S, susceptible; I, intermediate; R, resistant.

c XDR, extensively drug resistant. XDR isolates were defined as isolates not susceptible to ≥5 of the following six antipseudomonal agents or agent classes: ceftazidime or cefepime, meropenem or imipenem, piperacillin-tazobactam, ciprofloxacin, gentamicin or tobramycin, and colistin (only resistant isolates were included in the definition).

d MDR, multidrug resistant. MDR isolates were defined as isolates not susceptible to antipseudomonal agents from ≥3 different antimicrobial classes from the following list: ceftazidime or cefepime, meropenem or imipenem, piperacillin-tazobactam, ciprofloxacin, gentamicin or tobramycin, and colistin (only resistant isolates were included in the definition).

e NS, not susceptible.

**TABLE 3:** Cefiderocol MIC distributions for P. aeruginosa isolates with antimicrobial-resistant phenotypes

P. aeruginosa phenotype (no. of isolates)	No. of isolates (% tested) at cefiderocol MIC (μg/mL) of:											
	≤0.06	0.12	0.25	0.5	1	2	4	8	16	32	64	>64
All (1,050)	50 (4.8)	68 (6.5)	233 (22.2)	298 (28.4)	156 (14.9)	161 (15.3)	66 (6.3)	12 (1.1)	4 (0.4)	2 (0.2)		
XDR^*a*^ (235)	15 (6.4)	12 (5.1)	41 (17.4)	52 (22.1)	38 (16.2)	51 (21.7)	20 (8.5)	6 (2.6)				
MDR^*b*^ (771)	37 (4.8)	47 (6.1)	168 (21.8)	199 (25.8)	120 (15.6)	129 (16.7)	55 (7.1)	11 (1.4)	4 (0.5)	1 (0.1)		
Ceftolozane-tazobactam-NS^*c*^ (138)	5 (3.6)	5 (3.6)	14 (10.1)	24 (17.4)	37 (26.8)	28 (20.3)	19 (13.8)	5 (3.6)		1 (0.7)		
Ceftazidime-avibactam-R (283)	1 (0.4)	8 (2.8)	51 (18.0)	79 (27.9)	55 (19.4)	48 (17.0)	31 (11.0)	6 (2.1)	3 (1.1)	1 (0.4)		
Imipenem-relebactam-NS (234)	11 (4.7)	13 (5.6)	54 (23.1)	57 (24.4)	30 (12.8)	39 (16.7)	27 (11.5)	3 (1.3)				
Piperacillin-tazobactam-NS (739)	34 (4.6)	47 (6.4)	152 (20.6)	187 (25.3)	120 (16.2)	125 (16.9)	58 (7.8)	11 (1.5)	4 (0.5)	1 (0.1)		
Meropenem-NS (745)	35 (4.7)	37 (5.0)	173 (23.2)	202 (27.1)	112 (15.0)	117 (15.7)	52 (7.0)	12 (1.6)	4 (0.5)	1 (0.1)		
Imipenem-NS (726)	40 (5.5)	38 (5.2)	171 (23.6)	184 (25.3)	109 (15.0)	121 (16.7)	49 (6.7)	10 (1.4)	3 (0.4)	1 (0.1)		
Cefepime-NS (673)	32 (4.8)	43 (6.4)	141 (21.0)	168 (25.0)	111 (16.5)	111 (16.5)	51 (7.6)	10 (1.5)	4 (0.6)	2 (0.3)		
Ceftazidime-NS (666)	29 (4.4)	37 (5.6)	139 (20.9)	166 (24.9)	107 (16.1)	118 (17.7)	54 (8.1)	10 (1.5)	4 (0.6)	2 (0.3)		
Ciprofloxacin-NS (750)	42 (5.6)	44 (5.9)	165 (22.0)	207 (27.6)	111 (14.8)	118 (15.7)	49 (6.5)	8 (1.1)	4 (0.5)	2 (0.3)		
Gentamicin-NS (368)	25 (6.8)	21 (5.7)	73 (19.8)	97 (26.4)	53 (14.4)	68 (18.5)	25 (6.8)	6 (1.6)				
Tobramycin-NS (197)	10 (5.1)	10 (5.1)	42 (21.3)	56 (28.4)	26 (13.2)	35 (17.8)	13 (6.6)	5 (2.5)				
Colistin-NS (43)	2 (4.7)	3 (7.0)	6 (14.0)	10 (23.3)	10 (23.3)	7 (16.3)	4 (9.3)	1 (2.3)				

a XDR, extensively drug resistant. XDR isolates were defined as isolates not susceptible to ≥5 of the following six antipseudomonal agents or agent classes: ceftazidime or cefepime, meropenem or imipenem, piperacillin-tazobactam, ciprofloxacin, gentamicin or tobramycin, and colistin (only resistant isolates were included in the definition).

b MDR, multidrug resistant. MDR isolates were defined as isolates not susceptible to antipseudomonal agents from ≥3 different antimicrobial classes from the following list: ceftazidime or cefepime, meropenem or imipenem, piperacillin-tazobactam, ciprofloxacin, gentamicin or tobramycin, and colistin (only resistant isolates were included in the definition).

c NS, not susceptible.

Mushtaq et al. assessed the *in vitro* activity of cefiderocol versus 111 P. aeruginosa isolates selected to represent producers of metallo-beta-lactamases and guiana extended-spectrum beta-lactamase (GES) carbapenemases, as well as other resistance mechanisms (18). Overall, 86.5% of their isolates were susceptible to cefiderocol (18). Karlowsky et al. evaluated the *in vitro* activity of cefiderocol versus 7,700 P. aeruginosa clinical isolates obtained from laboratories in North America and Europe (2014 to 2019) (19). The MIC_90_ values of cefiderocol for the isolate subsets not susceptible to ceftazidime-avibactam (*n* = 477), ceftolozane-tazobactam (*n* = 463), and meropenem (*n* = 1,759) were 2 μg/mL, 2 μg/mL, and 1 μg/mL, respectively (19). These data and the results of the present study demonstrate that cefiderocol retains *in vitro* activity versus P. aeruginosa isolates with a diverse range of antimicrobial-resistant phenotypes. At present, there are limited clinical studies evaluating cefiderocol for the treatment of infections caused by antimicrobial-resistant P. aeruginosa (11–13, 20). The clinical and microbiological efficacy of cefiderocol was similar to the best available therapy for the treatment of patients with serious infections caused by carbapenem-resistant Gram-negative bacteria in an open-label trial (CREDIBLE-CR), although mortality was numerically higher among those in the cefiderocol arm (20). A subset of patients included in this trial had an infection caused by P. aeruginosa. Patients in that subset with a monomicrobial P. aeruginosa infection (all isolates were carbapenem resistant) showed no mortality difference. Further studies are needed to help define the role of this antimicrobial in clinical practice.

There are several limitations to this work that deserve attention. Molecular studies were not undertaken to determine the mechanisms of resistance to various antipseudomonal antimicrobials among the included isolates. Due to variability in the prevalence of antimicrobial resistance mechanisms in different geographic locations, it is possible that the results from this study may not be applicable to isolates from all regions (e.g., New Delhi metallo-beta lactamase [NDM]-producing bacteria are uncommon in Canada). We also did not evaluate the mechanisms conferring reduced susceptibility to cefiderocol in this study. Finally, as the isolates tested here were specifically selected due to their reduced susceptibility to various antipseudomonal antimicrobials, resistance rates for the different antimicrobials included on the testing panel may not be directly comparable.

In conclusion, cefiderocol was highly active *in vitro* (98.3% susceptible) against a selected collection of P. aeruginosa clinical isolates with beta-lactam and non-beta-lactam nonsusceptible phenotypes. Cefiderocol retained *in vitro* activity against the vast majority of XDR (97.4% susceptible) and MDR (97.9% susceptible) isolates, as well as isolates testing not susceptible to antimicrobials often reserved for the management of infections caused by antimicrobial-resistant pathogens (e.g., ceftolozane-tazobactam, ceftazidime-avibactam, and imipenem-relebactam). These *in vitro* data suggest that cefiderocol may be a treatment option for infections caused by highly antimicrobial-resistant P. aeruginosa, but further clinical studies are required.
